# The Role of Negative Emotions Pre- and Post-Implementation of Graphic Health Warnings: Longitudinal Evidence from South Korea

**DOI:** 10.3390/ijerph17155393

**Published:** 2020-07-27

**Authors:** Seon Min Lee, Seungwoo Chun, Jin Suk Lee

**Affiliations:** 1 Business School, Korea University, Seoul 02841, Korea; Se0n1ee@korea.ac.kr; 2 Dongguk Business School, Dongguk University, Seoul 04620, Korea; schun5@dongguk.edu

**Keywords:** Graphic Health Warnings (GHW), GHW policy, smoking cessation, negative emotions, perception of smoking risk

## Abstract

This longitudinal study investigated the factors that determine the effectiveness of graphic health warnings (GHWs) by comparing 246 South Korean smoker’s responses before and after the introduction of the country’s new tobacco control policy wherein GHWs were placed on all cigarette packaging. Even though introducing GHWs did not cause immediate changes in smokers’ intention to quit smoking or perception of smoking’s health risk, GHWs eventually motivated smokers to quit smoking when they experienced negative emotional responses to the newly introduced graphic warnings on cigarette packaging. More importantly, this study found that positive changes in smokers’ perceived risk associated with smoking due to the introduction of GHWs mediated a positive relationship between changes in smokers’ negative emotions (NE) from text-only warnings to graphic warnings and changes in their intention to quit smoking during the same period. Based on these results, the authors suggest that, for GHW policy to be more effective in motivating smoking cessation, the warnings need to convey images sufficiently unpleasant to induce negative emotional responses among smokers.

## 1. Introduction

Smoking is a well-known cause of preventable premature deaths [1] and public health risk. In 2015, the daily smoking rate of adult males in South Korea was 31.4%, which was one of the top countries in terms of the smoking rate among the 36 member countries of the Organization for Economic Cooperation and Development (OECD) [2]. The estimated cost of smoking prevalence in South Korea was approximately 1% of the country’s gross domestic product (GDP) [3]. On 23 December 2016, South Korea introduced their graphic health warning (GHW) anti-tobacco campaign. They unveiled 10 full-color pictorials along with text warnings that are mandated to cover at least 50% (i.e., 30% graphic and 20% text) of the front side of every cigarette pack (see Supplementary Table S1). Before GHWs were introduced, cigarette packs carried text warnings that covered only 20% of the pack’s front side. Ever since Iceland implemented black and white picture warnings in 1986, many countries, including Canada, Singapore, and Taiwan, have officially implemented GHWs on tobacco packaging, now in full color, as an effective tool to reduce national smoking rates [4,5]. The South Korean government expected that this policy would help to reduce the country’s smoking rates to the OECD member countries’ average smoking rate of 29% by 2020 [6].

Although many studies have examined the impact of various interventions on reduction in smoking and expansion of health consciousness, to the best of the authors’ knowledge, little research has directly examined why GHWs are effective in persuading smokers to quit. We focused on the role of negative emotions (NE) in activating the effects of GHWs on persuasion. Prior studies have suggested that people experience negative emotions when they view GHWs on tobacco packaging [7,8,9]. Negative emotions affect human cognitive activity and behaviors such as judgment, decision-making, and attitude [10,11]. We suggest that changes in negative emotional responses caused by viewing GHWs might increase their perception of the health risk of smoking, thereby persuading them to cease smoking. This research tests the prediction by means of a longitudinal study that was conducted during the introductory period of a new regulation requiring GHWs to appear on all cigarette packs sold in South Korea.

We conducted a quasi-experimental panel study, with individual panels held approximately one year apart, to estimate the effectiveness of GHW introduction—the national transformation of text-only warnings into graphic health warnings on cigarette packs in South Korea. The purpose of conducting this study was twofold: (1) to assess changes in smoker emotions, perceived smoking risk, and quitting behaviors before and after the introduction of GHW policy and (2) to understand the underlying mechanism of the effect of GHWs’ introduction on smokers’ quitting behavior.

Ethical approval for this study was granted by the Ethics Committee of Gachon University (project identification code: 1044396-201612-HR-094-01.)

## 2. Literature Review and Hypothesis Development

### 2.1. Various Interventions in Tobacco Control

Many countries take comprehensive interventions trying to reduce demand for tobacco products combining pricing (increasing price and tax of cigarettes) and non-pricing policies (smoking bans in public places, text and graphic health warnings, smoking cessation services, advertising regulations etc.) following the World Health Organization’s (WHO) recommendations [12]. First, cigarette price increases through higher taxation is considered to be one of the most effective strategies to reduce tobacco use and to motivate smoking cessation [13,14,15]. According to the World Bank [16], when the price of cigarettes was raised by 10%, tobacco consumption decreased by 4% in high-income countries, 8% in mid-to low-income countries, and the smoking rates has decreased by 2% and 4%, respectively. Second, non-pricing strategies including smoking bans in public places pursuits to reduce the smokers’ temptation as well as the public’s exposures to smoking. As the risks of second-hand smoke have been scientifically proven, countries have set and expanded non-smoking areas in accordance with the WHO Framework Convention on Tobacco Control’s guidelines [13]. Zhou et al. [17] showed that the implementations of this policy significantly impacts smoking cessation by a systematic review. They found that the higher the smoker’s dependence on nicotine and the less knowledge he or she has about tobacco, the more negative attitude one has toward tobacco policy and the policy of non-smoking areas is less effective. Bans on advertising, promoting and sponsorship of tobacco have the same purpose on tobacco control. Saffer and Chaloupka [18] found, by investigating the effect of tobacco advertising bans on tobacco consumption among 22 OECD countries. Most countries, especially developing countries, where anti-smoking policies were effective have used a comprehensive set of policies together rather than implementing only a specific single policy [18,19]. 

In similar vein, the combined use of strategies was effective in South Korea. In 2015, the Korean government increased the retail price of cigarettes, mainly due to higher taxation, about 80% higher than the previous price after a 10-year price freeze. The combination of this pricing policy with non-pricing policies such as an expansion of smoking-free areas from public places to insides of any buildings, restaurants, bars, and coffee shops increased smoking cessation from 7.2–9.9% [20].

### 2.2. Smoker Responses to the Implementation of Graphic Health Warning (GHW) Policy

As a second wave of tobacco control at the end of 2016, the South Korean government enforced mandatory GHWs on every cigarette pack sold which is known as one of the non-pricing strategies to reduce demand for tobacco products. Many studies have found evidence both for and against the effectiveness of GHWs. The predominant stream of research found evidence to support the assertion that GHWs are more effective in promoting smoking cessation than text-only warnings. The direct visual depiction of harmful health consequences (e.g., smoking-related throat cancer, skin discoloration, loss of lung function) causes smokers to pay more attention to warnings [21], prompts stronger cognitive and emotional reactions [22,23], increases smokers’ intention to quit (IQ) [24], increases non-smokers’ intention to avoid smoking [25,26], and generally reduces smoking behaviors [27]. Interestingly, many of the studies demonstrated that smoking rates were reduced in countries which implemented GHW policy, such as China, Canada, and the Netherlands [8,24,26,27,28,29]. However, another stream of research has reported that graphic warnings led to strong feelings of being threatened and maladaptive responses in smokers, with a range of effects including discounting the messages [30], displaying psychological reactance [31], and increasing intent to smoke [32]. ]. It is noticeable that no effect or negative effects from GHWs found in studies conducted in countries that had not yet adopted GHW policy such as the United States (US) [31,32]. For example, studies by Hammond et al. [28] and Sabbane et al. [32] demonstrated that GHWs worked well in Canada, but not as well in the US. However, Evans et al. [23] investigated US university students by establishing an environment in which they were naturally exposed to GHWs for four weeks and eventually discovered positive effects.

Based on such evidence, we suggest that GHWs will increase a smoker’s intent to quit smoking, negative emotions, and perception of smoking-related health risk when repeatedly exposed to GHWs. First, we propose that the introductions of GHWs might increase smokers’ intent to quit smoking. Most evidence that does not support GHWs’ positive effect on intent to quit is derived from experimental studies of countries in which GHW regulations had not yet been implemented. In such studies, research panels were asked questions about attitude and intent to quit after a one-time exposure to strong GHWs that may induce hostility. This experimental setting might be quite different from more realistic contexts in which individuals are exposed and react to GHWs. Cognitive reactance, the most common psychological mechanism explaining the boomerang effect of GHWs on smoking cessation, could be a smoker’s immediate response to a GHW to avoid the negative feelings induced by its graphic imagery. The acute and immediate response of avoidance due to the negative feelings aroused by GHWs might wane if smokers were repeatedly exposed to strong graphic images in everyday life. Indeed, Gibson et al. [33] found that a one-time exposure to strong graphic warnings in a laboratory setting was more effective than text warnings in increasing negative emotional responses in smokers but were not sufficient to increase their intent to quit. Their findings hinted at the possibility that the negative emotions produced by strong reactance were not enduring and were ultimately reduced in a real-world setting.

Second, we expect smokers to experience negative emotions when exposed to GHWs after the introduction of the policy. Graphic warnings were more effective in inducing emotional arousal [24,34] because the graphics depicted smoking-related diseases and damaged body organs by means of strong and sensational imagery [35]. GHWs have been shown to elicit negative emotions such as fear, disgust, worry, and guilt [7,8,9]. For example, Hammond et al. [8] discovered that GHWs elicited fear and disgust and examined the relationship between such emotions and quitting smoking. Netemeyer et al. [9] reported that when exposed to GHWs, smokers more often experienced disgust than non-smokers. In addition, they found that guilt can be elicited by GHWs. Therefore, we predict that the implementation of GHW policy might induce negative emotional responses in smokers.

Third, we suggest that smokers might realize the health risk of smoking from GHWs and feel vulnerable to these risks after the policy’s introduction. White et al. [36] discovered that GHWs increased the perceived risk of smoking (PRS) among Australian adolescents by comparing students before and after the introduction of the new GHWs. Hammond et al. [28] examined differences in the effectiveness of GHWs between four countries, namely, Canada, the United Kingdom (UK), Australia, and the US, between 2002–2005. They found that in Canada, where GHW policy had already been implemented for almost 20 years, smokers thought more about their health risk from smoking than smokers in the US, a country that uses text-only warnings. Interestingly, during the study period (i.e., in 2002) of the Hammond study, the UK introduced GHWs and it was found that British smokers considered health risk more seriously after the implementation of GHW policy than they did previously. Such results imply the existence of a linear relationship between time and GHWs’ effect on the perceived risk of smoking. Therefore, we suggest that the introduction of GHWs might enable smokers to realize the hazards of smoking to their health.

**Hypothesis 1** **(H1).** *Smokers’ intent to quit is higher after the introduction of GHWs than before.*

**Hypothesis 2** **(H2).** *Smokers’ negative emotions are higher after the introduction of GHWs than before.*

**Hypothesis 3** **(H3).** *Smokers’ perceived risk associated with smoking is higher after the introduction of GHWs than before.*

### 2.3. Negative Emotions, Perceived Smoking Risk, and Intent to Quit

GHWs elicit a greater level of negative emotion than text-only warnings [24,34] and induce various negative emotions including fear, disgust, worry, and guilt [7,8,9]. However, previous research has reported mixed findings, documenting both positive [8,37] and negative effects of this negative emotional response on quitting smoking [34,38,39]. Noticeably, those studies that reported supportive evidence were conducted in Canada many years after GHW policy was implemented. In other words, positive effect of the negative emotional responses to GHWs effect on quitting smoking could be valid only in cases in which smokers are repetitively exposed to GHWs in their daily lives [37]. Therefore, we predict that negative emotions from GHWs could have a dual impact with respect to time—negative emotional responses might not change smoker attitudes and smoking behavior in the short term, but, in the long term, repeated exposure to GHWs might eventually promote attempts to quit smoking. In other words, we focus on negative emotions as an important factor activating the effect of GHWs, but the effect is expected to take a certain length of time to appear. To test our predictions, we investigated how the GHWs’ introduction affected smoker emotions and how their emotions were associated with perceived risk of smoking and quitting behavior by comparing South Korean smokers’ reactions pre- and post-introduction of GHWs.

Negative emotions serve a dual role: as information and as a motivation [34,38,39]. The “affect-as-information” model states that people use negative feelings associated with a target to evaluate a target negatively [39]. In terms of this informational view, smokers often rely on their feelings as a source of judgement rather than deliberately evaluating the target’s objective features or attributes. In turn, these feelings affect their decision-making and behavior [40]. On the other hand, smokers could appraise the potential hazards of smoking in a more systematic way by using scientific realism when in a negative mood while they could be quite optimistic about estimating the possible hazards of smoking that might apply to themselves in a normal mood [41,42]. When exposed to GHWs stimuli, smokers might experience negative emotions that have a dual role. On the one hand, they might lead to the development of reactance if a smoker experiences an especially intense negative emotional response such as anger. In this case, the consequences might be rather counterproductive. However, a strong, acute, and intense negative emotional response does not last long enough to have a maximal effect, so when GHWs are implemented, repeated daily exposure to GHWs might eventually reduce reactance in smokers. Similar findings were reported by Cho et al. [37]. 

We suggest that the negative feelings aroused by GHWs might affect smokers’ recognition of the health risk associated with smoking to be more severe for two reasons. First, in line with the literature, intense negative emotions would act as information to make smokers to avoid or ignore GHWs immediately while less intense i.e., moderate to weak negative emotional responses might lead smokers to systematically elaborate about smoking hazards. Therefore, it might be helpful to increase the number of attempts to quit smoking by voluntarily finding additional reasons to avoid cigarettes. Second, when people experience negative emotions that are associated with a certain product or activity, they tend to evaluate the cigarettes or the activity of smoking negatively and perceive the associated risk to be more important and, accordingly, the associated benefits to be less important [43]. Evans et al. [23] found that GHWs caused negative emotional responses that increased perception of the risk of smoking. In addition, Netemeyer et al. [9] showed that the fear elicited by GHWs mediated the relationship between GHWs and adolescent smokers’ current consideration of smoking. The more frightened those adolescent smokers became when exposed to GHWs, the more they reflected on the consequences of their own smoking, such as the negative health effects of smoking on themselves and others. The results of this study imply that negative emotions from GHWs might motivate smokers to perceive the risk of smoking through a more realistic lens and consequently increase their perception of the risk of smoking. Therefore, due to GHWs’ introduction, the negative emotional responses from repeated exposures to GHWs might increase Korean smokers’ recognition of the risk associated with smoking.

**Hypothesis 4** **(H4).** *Before and after GHWs introduction, changes in negative emotions are positively related to changes in perceived health risks of smoking.*

Next, we suggest that changes in negative emotions and perceived smoking risk might play a critical role in producing behavioral changes in smokers after GHW policy is introduced. Evidence supports a positive relationship between negative emotions and intent to quit smoking [9,24]. Kees et al. [24] reported a mediating role of fear between GHWs and intent to quit smoking. By analyzing the mediating effect of fear, disgust, and guilt in the relationship, Netemeyer et al. [9] showed that fear performs mainly a mediating role. However, worry also mediated GHWs’ effect [22]. In addition, neurological studies showed that, among smokers, more emotionally arousing pictorial warnings caused a stronger activation of the brain regions associated with decision-making and memory formation [44,45]. As found in functional magnetic resonance imaging (fMRI) research on smoking cessation advertisements [46], strong brain activation prompted by graphic cigarette warnings predicts a decrease in smoking [47]. These studies focused on the negative emotions elicited by GHWs and confirmed their mediating effects.

Based on previous studies that established that a variety of negative emotions are critical variables, we suggest that the overall negative emotion elicited by GHWs’ introduction affects smokers’ intent to quit smoking.

**Hypothesis 5** **(H5).** *Before and after GHWs introduction, changes in negative emotions are related to changes in intent to quit.*

Several studies have noticed the dual function of negative emotions: informational as well as motivational [39,48]. People often diagnose their current situation as being problematic when they experience negative emotions and, at the same time, the negative emotions stimulate analytical processing to solve the problem [39]. The cognition most associated with negative emotions is risk perception [48,49]. Since the perception of risk would be amplified by fear [49], negative emotions lead people to perceive the hazards of smoking and estimated risk associated with smoking [22,23]. Based on the role of analytical thinking, negative emotions elicited by GHWs might induce motivation to contemplate the maladaptive consequences of smoking. Similarly, the extended parallel process model [50] emphasized the estimated threat as a critical determinant of changing behavior in order for advertisements using fear appeal to be effective. When people more intensely perceive risk after viewing fear-appeal advertising, their behavior changes. In addition, in some experimental studies with self-reported measures, negative affective reactions provoked by warnings cued further processing of warning information and ultimately, motivated smoking cessation [23,38]. Indeed, some studies found a dual mediating role of negative emotions and risk perception of GHWs’ effect on smokers’ intent to quit [7,22,23,24]. Emery et al. [22] showed that GHWs have a positive effect on intent to quit smoking by means of increasing worry as well as risk perception.

In line with the preceding rationale, we suggest that the perceived changes of risk level after GHWs introduction would mediate the effect of negative emotions on smoker intent to quit smoking.

**Hypothesis 6** **(H6).** *Both before and after GHWs’ introduction, perceived risk associated with smoking mediates the effect of negative emotions on intent to quit.*

## 3. Methods

### 3.1. Procedure and Panels

Only current smokers were eligible to participate in the surveys. The International Tobacco Control (ITC) survey [51] used three criteria to classify individuals as smokers: if they had smoked more than 100 cigarettes (i.e., five packs) over their lifetimes; if they had smoked more than once during the preceding month; and if they were over 19 years of age. Smokers were recruited to participate in two opt-in panel surveys—one before and one after the legal implementation of GHWs—conducted by a marketing research company in Korea. The pre-introduction survey was conducted with 368 smokers during the first week of December 2016 (05.12.2016–11.12.2016). The introduction of GHWs began on 23 December 2016, but it took two or three months for cigarette packaging with GHWs to be fully available at local retailers throughout the country.

All panels were adult smokers over 20 years old who had smoked more than 100 cigarettes in their lifetime. The majority (84.1%) of respondents reported that they had last smoked within the preceding 24 h; 10.6% of participants had smoked between the preceding 24 h and the preceding week, and 5.3% had smoked between the preceding week and the preceding month.

The sample was recruited from different age groups and regions, and only those who participated in both the pre- and post-introduction surveys were analyzed for the study. Of the pre-introduction participants, only 246 returned to take part in the second survey that was conducted during the first week of October 2017 (02.10.2017–08.10.2017) (i.e., a return rate of 66.85%). We analyzed 246 pieces of data of those who participated in both surveys. We stratified the sample by age—the average age was 40.48 with a standard deviation (SD) of 10.88–19.5% of the sample in their 20s, 25.2% in their 30s, 28.0% in their 40s, and 27.2% in their 50s, as well as by gender (52.8% men, 47.2% women). The majority of the respondents were city dwellers (96.7%). They spent about 163 dollars ($) (equivalent to 174,100 won) per month on cigarettes purchases that accounted for 3.91% of their average monthly earnings ($4160, or 4.45 million won). On average, they smoked 11.34 cigarettes per day. Most respondents (i.e., 65.4%) considered themselves to be addicted to smoking and only 6.5% said that they were not addicted. In support of this, 57.3% of the survey participants reported that they had smoked every day during the preceding month.

### 3.2. Measures

Each participant in this study was invited to take part in the online survey twice: one month before the introduction of GHWs (i.e., pre-introduction) and 10 months after their introduction (i.e., post-introduction). The research firm sent email invitations with individual survey links to track responses.

#### 3.2.1. Negative Emotions (NE)

The extent of six negative emotions —worry, guilt, disgust, sadness, regret, and anger—from the current warnings on cigarette packaging were assessed with the items adapted from Gibson et al. [33]. We used the same questions for both the pre- and post-introduction surveys but, due to the study design, the targets that these items measured differed: they were text-only warnings at the pre-introduction survey and GHWs at the post-introduction survey. Each item was judged using a seven-point Likert scale, with anchors of one (least likely to feel) to seven (most likely to feel). The summed scores produced satisfactory Cronbach’s alpha coefficients for both surveys: text-only warnings during the pre-introduction period (α = 0.91) and GHWs during the post-introduction period (α = 0.95).

#### 3.2.2. Perceived Risk of Smoking (PRS)

A panel of participants used a seven-point Likert scale ranging from one (strongly disagree) to seven (strongly agree) to indicate the extent to which they agreed with two statements to measure perception of the health risk of cigarette smoking: (1) smoking is harmful to health (e.g., severity) and (2) smokers’ probability of death is much higher than that of non-smokers (e.g., vulnerability). These values were summed to calculate a pre-introduction perceived risk score (α = 0.78) and a post-introduction perceived risk score (α = 0.84).

#### 3.2.3. Intent to Quit Smoking (IQ)

A two-item scale was used to assess the degree to which a panel of participants was willing to quit smoking [52]. The participants used a seven-point Likert scale ranging from one (extremely unlikely) to seven (extremely likely) to indicate the degree to which they might make a plan to quit smoking within the next month or within the next six months. The items were summed, rather than averaged, to create, for each participant, a total pre-introduction score (α = 0.80) and post- introduction score (α = 0.81) since the two items were significantly correlated at a 5% significance level (*r* = 0.67 for pre-introduction and *r* = 0.69 for post-introduction).

## 4. Results

### 4.1. Attrition Analysis

To detect attrition bias caused by extinction of 122 participants (from 368 in the pre-introduction survey to 246 in the post-introduction survey), we conducted *t* tests to compare those subjects responding to pre- and post-introduction surveys with those responding to only the pre-introduction survey. There were no significant differences in the means of primary variables of the study such as pre-NE (*t* = −1.85, *p* > 0.05), pre−PRS (*t* = −0.47, *p* > 0.05), pre-IQ (*t* = −0.32, *p* > 0.05), average number of cigarettes per day (*t* = 1.79, *p* > 0.05) and sex (chi-square = 3.409, *p* > 0.05). However, only age difference was significant (*t* = −2.08, *p* < 0.05). The average age of participants only in pre-introduction survey (37.95 years old) was 2.52 years lower than that of participants in both surveys (40.48 years old). Cross tabulation analysis of drop-out and no drop-out groups and age groups showed that participants in their 20s (44.8%) dropped out relatively more than 30 s (32.6%), 40 s (25.8%), and 50 s (30.2%). To examine the difference between 20 s drop-outs and 20s non-dropouts, we conducted *t* tests on key variables. No significant differences were found in pre-NE (*t* = −0.39, *p* > 0.05), pre-PRS (*t* = 0.78, *p* > 0.05), pre-IQ (*t* = −0.46, *p* > 0.05). While attrition was not random with age, it was not related to the primary variables on which this study focuses. Overall, the results indicated no evidence of attribution bias in this study.

### 4.2. Pre-Introduction and Post-Introduction Differences in Negative Emotions, Subjective Risk, and Intent to Quit Smoking: H1, H2 and H3 

A series of paired two-sample *t* tests was conducted to compare differences in the negative emotional responses to type of warning, perceived risk of smoking, and intent to quit smoking pre- and post-introduction of GHWs. As shown in Table 1, negative emotions increased significantly from a mean score of 3.84 (SD = 1.40) for the pre-introduction survey to a mean score of 4.50 (SD = 1.27) for the post-introduction survey (*t* (245) = −7.54, *p* <.001). However, neither mean values changed for the perceived risk of smoking (pre-introduction M = 5.35 (SD = 1.20) vs. post-introduction M = 5.30 (SD = 1.23); *t* (245) = 0.59, *p* = 0.55) nor for intent to quit smoking (pre-introduction M = 3.02 (SD = 1.11) vs. post-introduction M = 2.96 (SD = 1.12); *t* (245) = 0.87, *p* = 0.38) which showed a statistically significant difference due to the introduction of GHWs. Thus, the prediction for the change in negative emotions due to GHWs’ introduction, Hypothesis 2, was supported, but the other hypotheses were not.

The findings show that respondents were indeed frightened or felt uncomfortable after the GHWs were introduced. However, the introduction of GHWs did not have an impact on respondents’ tendency to give them more thought or change their willingness to quit smoking.

### 4.3. Testing the Longitudinal Hypotheses: H4 and H5 

A simple regression analysis was used to test the longitudinal association between the positive mean changes in negative emotions between text-only and graphic warnings on cigarette packs (∆NE) and the increased level of perceived health risk associated with smoking (∆PRS) during the same period. The results indicated that changes of negative emotions significantly predicted changes of perceived risk of smoking (β = 0.17, *t* = 2.75, *p* < 0.05). In addition, we entered pre-introduction perceived risk of smoking (pre-PRS) and pre-introduction negative emotional responses to text warnings (pre-NE) as control variables to the first regression model. The results indicate that the effect of respondents’ negative emotional changes on their risk perception changes became larger (β = 0.36, *t* = 5.37, *p* < 0.001) after controlling for the pre-introduction effect of negative emotions toward text-only warnings (*t* = 3.66, *p* < 0.001) and pre-introduction perceived risk of smoking (*t* = −9.47, *p* < 0.001; see Table 2). This evidence confirms Hypothesis 4.

The similar procedure of analysis was conducted to test Hypothesis 5. The results showed that the changes of negative emotions toward cigarette package warning between pre- and post-introduction of GHWs was significantly associated with the changes of intention to quit in both the simple regression (β = 0.12, *t* = 2.79, *p* < 0.05) and the multiple regression (β = 0.19, *t* = 2.79, *p* < 0.05) conditions. Thus, Hypothesis 5 was supported. Details of these results are shown in Table 2.

These findings demonstrate that participants perceived a higher level of health risk of smoking since they experienced more negative emotions from GHWs over time. The increased level of negative emotional responses also longitudinally increased respondents’ intentions to quit smoking.

### 4.4. Testing the Mediation Hypothesis: H6

**Figure 1:**
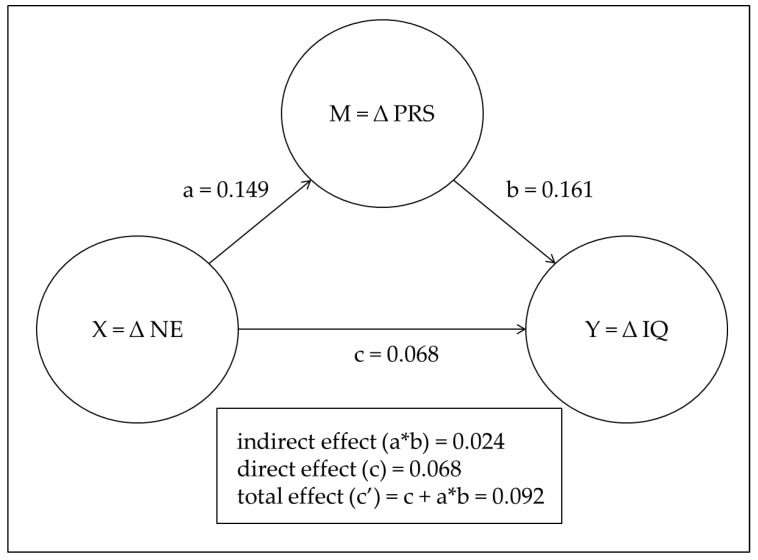
Results of mediation analysis based on bootstrapping.

We predicted that the influence of enhanced negative emotional response on increased level of quitting intention would be mediated by changes in the perceived level of smoking risk before and after GHWs were introduced. To test mediating effect, we conducted Hayes’ PROCESS macro model 4 with ∆IQ as a dependent variable, ∆NE as an independent variable, and ∆PRS as a mediating variable. Recently in area of psychology research, this PPOCESS macro model based on the bootstrapping test has been widely used for the mediation effect as a better way to supplement the limitations of Baron and Kenny’s mediation analysis [53]. As shown as Figure 1, a bootstrap analysis [54] revealed that the increased level of perceived smoking risk mediated the association between mean changes in negative emotions and mean changes in intent to quit (indirect effect = 0.024, SE = 0.013, 95% CI = (0.005, 0.054)). The direct effect between mean changes in negative emotions and mean changes in intent to quit was not significant (direct effect = 0.068, SE = 0.047, 95% CI = (−0.025, 0.161)). 

These findings show that, despite the insignificant direct association, participants reported an increased intention to quit smoking when they experienced a more negative emotional response from the newly introduced GHWs because they perceived a higher risk associated with smoking than before their introduction.

## 5. Discussion

This study investigated two issues related to the introduction of GHW policy in South Korea. The first issue was whether mandatory GHWs would change smokers’ emotions, perception of risk, and intention to quit immediately or after the passage of a certain time. The second issue concerned testing the mediating role of elaborated thought about smoking risk due to the negative emotions generated by GHWs and the effect of the policy’s introduction on quitting attempts among adult cigarette smokers.

This longitudinal study, which compares variables before and after the implementation of GHWs in South Korea, demonstrated that GHWs’ introduction had a direct influence on negative emotions. This supports previous research findings that graphic warnings elicit negative emotional responses [24,55]. However, there were no direct effects of GHWs’ introduction on smokers’ intent to quit or smoking risk perception, which differs from the results in previous studies. Longitudinal studies conducted in countries that had previously implemented GHW policy found that GHWs directly affect smoker perception of risk [28,36] and intent to quit smoking [8,24,26,27,28]. However, in this study, the negative emotions elicited because of repeated exposure to GHWs indirectly increased smoker intent to quit by means of GHWs’ higher level of perception of smoking risk. When exposed to a cigarette pack every day after the GHW policy’s introduction, smokers’ negative emotions toward the graphic warnings might be accompanied by a presumably more accurate estimate of smoking risk than previously, which mediates the direct relationship between GHWs’ introduction and intent to quit smoking. Although GHWs’ introduction did not directly increase intent to quit smoking, it increased negative emotional responses that caused smokers to be more vividly aware of their own vulnerability to the potential risk of smoking. Noticeably, these negative feelings were powerful enough to directly influence smokers’ intent to quit. These emotional and perceptual changes caused by repeated exposure to GHWs in daily life might promote self-directed and voluntary behavioral changes to conform to GHWs’ persuasive messages. In other words, GHWs’ introduction was effective in motivating smokers to accept the graphic warnings’ information and persuading smokers to voluntarily cease smoking.

## 6. Conclusions

This study contributes to existing literature on the effects of GHWs in a couple of ways. First, our findings shed light on how and why the effects of GHWs might work by examining the latent rather than the immediate effects of GHWs on changes in smoker emotions, perceived risk of smoking, and quitting behavior. To the best of our knowledge, this is the first longitudinal study to demonstrate the role of perceived risk of smoking to motivate smokers to attempt smoking cessation since they experience relatively more negative emotions toward GHWs than text-only warnings. While previous longitudinal studies used different samples or did not apply the same panels for analysis of the effects of GHWs’ introduction [6,28], the current study investigated the effects by comparing the same panel’s responses before and after the introduction of GHWs. This is a more rigorous way to verify the effects of GHWs. Second, we examined the overall role of the negative emotional responses elicited by GHWs in their effect on smoker intent to quit smoking. Previous studies investigated how discrete negative emotions—such as fear and disgust—work in increasing smoking cessation behavior in experimental settings [55] or how an advertisement with fear appeal works [50,56]. However, recent research on the effects of GHWs’ emotional responses on information processing [34,37,40] has examined its effect by integrating negative emotions. Therefore, we demonstrated that integrated negative feelings, not one discrete negative emotion, might work in the same way. The experience of overall negative feelings increased smoker intent to quit smoking by means of increasing perceived smoking risk in terms of severity and vulnerability, which worked as a critical driver in the effect of GHWs on smoking cessation. This study expands the depth of understanding of the effects of the negative emotions that are experienced as a result of GHWs.

In addition, this research enriches fear-appeal literature by resolving two contradictory findings. Traditional fear-appeal literature, such as the extended parallel process model that emphasizes the role of fear and perception of risk, has suggested that a mild level of fear works best, but extremely strong fear might backfire on GHWs’ effects [50]. However, recent studies have demonstrated a linear relationship between the persuasion effect and the strength of fear appeal [56]. These studies found that a strong fear appeal in advertising might be effective in persuading viewers to change their attitude and increase their intent to purchase. Our findings provide an alternative explanation of why some applications of strong fear appeal are successful while others are not. It is possible that strong fear appeal might be effective only when individuals must accept it and are repeatedly exposed to it. In this longitudinal study, smokers should accept the general negative feelings, including fear, from GHWs on cigarette packs as a matter of daily routine. Then, the changes in negative emotions pre- and post-introduction of GHW policy actually led to an increase in intent to quit smoking by means of perceived health risk of smoking. Therefore, we suggest that, in order for fear appeal to be successful, the stimulus should be exposed for a sufficiently long duration to remind targets of the relevant risk associated with fear.

The current study has important political implications for the sustainability of public health interventions. The introduction of GHW policy in South Korea changed tobacco warnings from text-only to a mandatory graphic image accompanying text warnings on cigarette packaging. A longitudinal field study conducted to compare smoker responses to tobacco warnings before and after this transformational policy implementation found GHW labels effectively persuade smokers both emotionally and cognitively by discouraging them from continuing to smoke. Repeated exposure to GHWs induced smokers to experience negative emotional responses that led them to more accurately estimate the hazards of smoking which, in turn, increased their intent to quit smoking. Although smokers might cognitively block threatening messages to control their feelings of fear, they regulated their misconduct (i.e., smoking) to mitigate the personal danger that the behavior might cause over the long term.

However, the current study has several limitations that require further research. First, these findings should be interpreted with caution, since only the South Korean case was tested for the effectiveness of GHW policy on promoting smoking cessation vis-à-vis quitting attempts. Further research should investigate another country to validate these findings. For example, the US Food and Drug Administration (FDA) announced that a new GHWs policy will be implemented in cigarette packages and advertisements in June 2021 [57]. Second, in addition to the effectiveness of GHWs’ introduction, as suggested in this study, the possibility exists that other variables such as smoking bans, prices increase, and media campaigns are also effective. Difficulty to control all extraneous variables is one limitation of longitudinal studies. However, this longitudinal study meets the key requirements of causal relation, association, and temporality, although alternative explanations cannot be ruled out. Therefore, these findings are sufficiently meaningful [6]. Third, the effectiveness of GHWs might be driven by the particular, cultural risk beliefs specifically targeted by the warnings implemented in South Korea (e.g., impotence) rather than the general health risk of smoking. However, it still remains unclear which type of risk perception is most effective in reducing intention to smoke. Fourth, another line of future research might explore a potential reversal in directional causation with the introduction of GHWs. It is possible that the changes in risk perception and quitting intentions might also drive negative emotional responses. Last, the sample size of this study is somewhat smaller than those of prior longitudinal studies. Our study is a comparative study of one sample group’s responses before and after the introduction of GHWs. In the area of GHW research, due to the difficulty of collecting and maintaining samples, studies that directly compare the responses of a single sample were rarely conducted. Therefore, despite the small sample size, our findings nonetheless contribute to research on tobacco packaging GHWs.

## Figures and Tables

**Table 1:** Results of paired sample *t* tests.

Variables		Mean	Standard Deviation (SD)	*t*	*p*
NE	Pre	3.84	1.40	− 7.54	0.00
	Post	4.50	1.27		
PRS	Pre	5.35	1.20	0.59	0.55
	Post	5.30	1.23		
IQ	Pre	3.02	1.11	0.87	0.38
	Post	2.96	1.12		

Note: NE = negative emotions, PRS = perceived risk of smoking, IQ = intention to quit smoking, Pre = before the introduction of the policy; Post = after the introduction of the policy.

**Table 2:** Results of testing for longitudinal effects.

Test Model	DV	IVs	β	*t*	*p*	R^2^	F (*p*)
∆NE → ∆PRS	∆PRS	∆NE	0.17	2.75	0.01	0.03	7.57 (0.01)
		∆NE	0.36	5.37	0.00	0.30	34.20 (0.00)
		Pre-NE	0.25	3.66	0.00		
		Pre-PRS	−0.53	−9.47	0.00		
∆NE → ∆IQ	∆IQ	∆NE	0.12	1.95	0.05	0.02	3.80 (0.05)
		∆NE	0.19	2.79	0.01	0.24	25.05 (0.00)
		Pre−NE	0.20	2.70	0.01		
		Pre-IQ	−0.50	−8.38	0.00		

Note: NE = negative emotions, PRS = perceived risk of smoking, IQ = intention to quit smoking.

## Supplementary Materials

The following are available online at https://www.mdpi.com/1660-4601/17/15/5393/s1, Table S1: Ten types of graphic warning used on cigarette packages.
