# Supplementary material for: The Role of Negative Emotions Pre- and Post-Implementation of Graphic Health Warnings: Longitudinal Evidence from South Korea
Source: Int J Environ Res Public Health. 2020 Jul 27;17(15):5393. doi: 10.3390/ijerph17155393 (PMC7432201; doi:10.3390/ijerph17155393)
Supplement: Supplementary file 1 [file ijerph-17-05393-s001.pdf]

**Supplementary Table S1:** Ten types of graphic warning used on cigarette packages.

| **Types** | Graphic Warnings (on the top of package) | Text Warnings | | |
|---|---|---|---|---|
| | | Front (Top) | Back | Side |
| 1. Lung cancer | 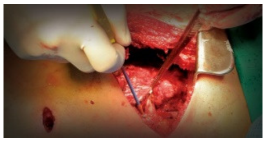 | Smoking causes lung cancer. Tobacco Quit line 1544-9030 | | |
| 2. Throat cancer | 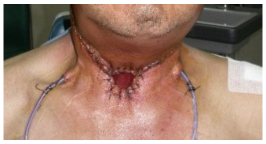 | Smoking causes throat cancer. Tobacco Quit line 1544-9030 | | |
| 3. Mouth cancer | 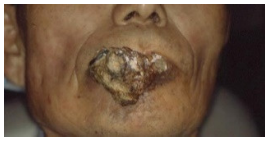 | Smoking causes mouth cancer. Tobacco Quit line 1544-9030 | | |
| 4. Heart disease | 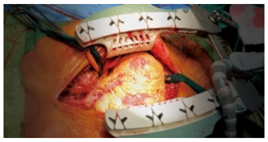 | Smoking causes heart disease. Tobacco Quit line 1544-9030 | | |
| 5. Cerebral stroke | 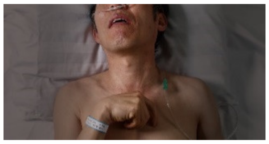 | Smoking causes the stroke. Tobacco Quit line 1544-9030 | Cigarette smoke contains carcinogenic substances such as naphthylamine, arsenic, benzene, arsenic, rides, arsenic, cadmium, etc.<br><br>Tobacco Quit line 1544-9030 | The amounts of tar intake depend on the smoking habits |
| 6. Secondhand smoking | 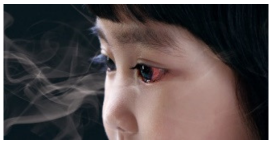 | Secondhand smoking harms children. Tobacco Quit line 1544-9030 | | |
| 7. Smoking during pregnancy | 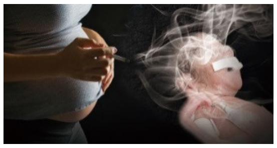 | Smoking during pregnancy has cause birth defects and miscarriage. Tobacco Quit line 1544-9030 | | |
| 8. Sexual dysfunction | 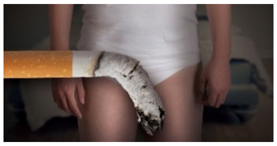 | Smoking causes erectile dysfunction. Tobacco Quit line 1544-9030 | | |
| 9. Skin aging | 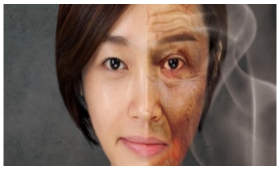 | Cigarette smoke causes skin pigmentation and vaporization. Tobacco Quit line 1544-9030 | | |

| | | |
|---|---|---|
| 10. Early death | 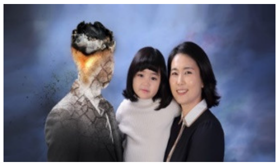 | Smoking causes the decline of average life span.<br>Tobacco Quit line 1544-9030 |

*Source*: A press release from the Ministry of Health and Welfare, Republic of Korea (22 June 2016).
